# Supplementary material for: Chlorination of Schistosoma mansoni cercariae
Source: PLoS Negl Trop Dis. 2020 Aug 21;14(8):e0008665. doi: 10.1371/journal.pntd.0008665 (PMC7467251; doi:10.1371/journal.pntd.0008665)
Supplement: Attachment — Submitted filename: 20200722 - Reviewer response.docx [file pntd.0008665.s001.docx]

Department of Civil and Environmental Engineering

Imperial College London

Skempton Building

South Kensington Campus

Exhibition Road

Tel: +44 (0)772 491 5446

[laura.braun13@imperial.ac.uk](mailto:laura.braun13@imperial.ac.uk)
https://www.imperial.ac.uk/people/laura.braun13

22/07/2020	**Laura Braun** BEng MSc

**Re: Revisions to PNTD-D-20-00846 “Chlorination of Schistosoma mansoni cercariae” (Braun *et al.*)**

Dear Dr. Häberlein and Prof. Zhou,

We thank you and the external reviewers for your valuable and helpful comments on our manuscript entitled “Chlorination of Schistosoma mansoni cercariae” (#PNTD-D-20-00846). We have incorporated your suggestions and revised the paper accordingly.

Attached is a summary of the feedback from the three peer-reviewers, along with our comments and revisions made. The revised manuscript with tracked changes is also enclosed.

Thank you again for considering our paper for publication in *PLoS Neglected Tropical Diseases*. We look forward to your final decision. Please do not hesitate to contact us should you have any further questions or comments.

Sincerely,

Laura Braun^1^, Yasinta Sylivester^2^, Meseret Dessalegne^3^, Muluwork Maru^3^, Fiona Allan^4^, Feleke Zewge^3^, Aidan M Emery^4^, Safari Kinunghi^2^, Michael R Templeton^1^

^1^ Department of Civil and Environmental Engineering, South Kensington Campus, Imperial College London, London SW7 2AZ, United Kingdom

^2^ National Institute for Medical Research, Mwanza Center, Isamilo Street, Ilemela, Mwanza, Tanzania

^3^ College of Natural and Computational Sciences, Addis Ababa University, Arat Kilo, Addis Ababa, Ethiopia

^4^ Wolfson Wellcome Biomedical Laboratories, Department of Life Sciences, Natural History Museum, Cromwell Road, London SW7 5BD, United Kingdom

| **Reviewers’ comments** | **Authors’ Response** | **Lines** |
| --- | --- | --- |
| It is considered that temperatures in Tanzania and elsewhere are sometimes below 20 degrees Celsius, the schistosomiasis cercariae will survive longer at low temperature and have better activity. It is of great significance to test the killing conditions of cercariae at low temperature. It is recommended to add the CT value of water chlorination at a lower temperature if possible. | Although air temperatures in schistosomiasis endemic regions fluctuate below our tested range of 20-27°C, water temperatures rarely drop below this. For example, in Lake Victoria the water temperature ranges between 23.5 - 29.0 °C [1, 2]. *Biomphalaria glabrata* snails which carry *Schistosoma mansoni* have been shown to die if kept at temperatures below 16°C [3]. In case the water is stored in a tank and allowed to cool to air temperature (<20°C), the required CT value would only be slightly higher than that at 20°C as our results show that temperature has a small impact on the CT value (1°C drop in temperature increases the CT value by 0.42 mg·min/l), unlike pH which affects the CT value more significantly (increase of 0.5 pH increases CT by 3.25 mg·min/l). Therefore, we feel that the range of 20-27°C is sufficient for developing these chlorination recommendations.  This clarification has been added to the manuscript. | 125-129 |
| For drinking water standards, the final chlorine residue is an issue to consider. It is recommended that the study give the result of residual chlorine concentration after water treatment. Whether it is necessary to use chlorine removal before using the treated water, and what kind of dechlorination operation is appropriate. | The free chlorine residuals achieved with our recommended CT values are within the WHO recommended range for drinking water.  Also, treated cercaria water in the context of this study is not intended for drinking (rather, it is for water contact activities, e.g. washing laundry, bathing), especially as the recommended CT values may not inactivate water-borne pathogens present in the water. Therefore, de-chlorination is not needed and should be discouraged as the free chlorine residual provides protection from recontamination.  Two sentences about dechlorination have been added to the manuscript. | 225-229 |
| Section beginning on line 333. The comments on the health-relevance of 2 log10 reduction as a normative target for treatment are most welcome. This may be an arbitrary benchmark. I do not really understand this argument:  “… water samples are unlikely to contain as high concentrations of cercariae as tested in our experiments (2 cercariae/ml). Therefore, a lower log inactivation may be sufficient.”  The authors rightly state just above that “water should be completely free of cercariae” (lines 338 and 339). The relevance of the 2 log reduction target, in terms of exposure, is unclear, since one cercaria = one schistosome and volume is relevant here because exposure is to volumes of water. Let’s take 1000 L of water. We add in 1000 cercariae, so 1 cercaria per L (far less concentrated than the experimental conditions of 2 cercariae per ml). We dose this to achieve 2 log reduction, according to the recommendations here. That leaves 10 cercaria in the volume. Would that produce any exposure risk? Would the risk be more tolerable, and by what standard? I’m grappling with what conclusions we can draw about the likelihood of the recommended treatment to reduce risk in endemic settings. Can the authors present a mechanistic argument for why a 2 log reduction is sufficient or even, as they suggest, conservative? Certainly fewer cercariae would be better than more, but how do we know this would be “good enough”, and how is “good enough” defined? | This is a very valid point. Unlike for bacteria or viruses, there is no standard log reduction that should be achieved to reduce schistosomiasis infection risk. A 2-log_10_ inactivation of cercaria was chosen for two reasons: Cercaria concentration in natural water is assumed to be <1 cercaria/100L, based on literature [4, 5]. Therefore, a 2-log_10_ inactivation would render water volumes up to 10,000 L safe (i.e. 10,000 L with a cercaria concentration of <1 cercaria/100L would lead to <100 cercariae. A 2-log inactivation should therefore remove all cercariae). Of course, the cercaria concentration varies significantly in water depending on the distance to shedding snails, among many other factors (e.g. water body type, season, flow rate, vegetation, number of snails, location in freshwater body etc) [4]. For this reason, water intended for treatment should always be abstracted away from vegetation/snail habitats to minimise cercaria numbers. The other reason for choosing 2-log_10_ is that it is difficult to achieve higher inactivation in the lab. Cercaria density and time required to count live/dead cercariae are limiting factors. Larger volumes of water could be tested, but this would make the viability assessment less accurate as it would take longer to count live/dead cercariae using our experimental protocol.  Overall, it is therefore difficult to know if a 2-log_10_ inactivation really is good enough, or too conservative. Based on the limited cercariometry data available, the fact that we only used fresh cercariae, and that movement was used as a viability measure, we are confident that the 2-log_10_ inactivation of cercariae will reduce the risk of infection. Our recommendation is also based on the worst-case pH and temperature, and the log reduction would be higher under more favourable chlorination conditions (higher temperature, lower pH). A decrease in the number of cercariae coming into contact with the host results in decreased infection and reduced worm number. The biggest risk associated with water treatment is that people do not use the treated water, and thereby expose themselves to much greater concentrations of viable cercariae than if they used water treated to a 2-log_10_ inactivation.  This has been clarified in the manuscript. | 354-368 |
| Line 27. Add log base. From the text, it’s clear this is log10, but should be specified. | Changed | 25, 27 |
| Line 349 and beyond. It should be acknowledged that most chlorine available in endemic settings would be of uncertain concentration to most users. Therefore the problem of measuring chlorine remains. CT values are valuable, but doses can only be shown to meet the target if we know what concentration of chlorine we have to begin with. Household bleach comes in a variety of concentrations and cannot be assumed in most settings. Empirical calibration of the dose, measured by a pool tester (at least), is probably the most feasible option in most settings. | Information regarding the instability of chlorine and variation in concentration has been added, along with a sentence that the chlorine concentration should be measured using a testing kit, if available. The example in lines 378-381 is for a certain concentration of household bleach. A sentence has been added that this example could be used to linearly scale the required dose to their concentration. E.g. a bleach concentration of 1% instead of 3% requires three times the dose. | 381-389 |
| This study investigates the effectiveness of chlorination against S. mansoni cercariae. Chlorination. The experiments indicate that S. mansoni cercariae can be inactivated up to 2-log with a CT value of 26 mg·min/l at pH 7.5 and 20°C, though adding in a safety factor to account for uncertainties would lead to a recommended CT value of 30 mg·min/l. It means that S. mansoni cercariae are sensitive to chlorine, and higher chlorine CT values are required with higher pH and lower temperature. The paper reads well, while important preliminary findings. One comment–in Discussion, the results presented here can be used as the basis for water treatment guidelines for S. mansoni infested water. How would the author suggest this treatment would be carried out? Because the results did not find a significant difference in chlorine sensitivities of laboratory-grown and field cercariae. | Indeed, the results indicate that there is no significant difference between lab-grown and field cercariae, and we therefore draw the conclusion that lab-data is valid for field settings. The recommended CT value of 30 mg·min/l for 2-log_10_ inactivation of cercariae holds for both lab and field settings. The treatment could be carried out in any way that meets the CT value, for example with a free chlorine residual of 1 mg/L after 30 minutes, as suggested in the paper. There are numerous different ways of dosing chlorine that vary with equipment and resource availability (e.g. chemical dosing pump, or manual dosing and stirring). This has been added to the manuscript. Lines 390-398 outline how the water could be treated, but the method will be very context specific. | 74-75  395-396 |

Other changes:

- Additional references have been added
- An ethics statement has been added to the methods section
- The acknowledgments have been updated to include Dr. Matt Berriman
- The ethics statement has been updated to include information on maintaining the lifecycle
- Other small word changes have been made to improve the paper, as shown by the tracked changes in the manuscript

References:

1.	Yin, X. and S.E. Nicholson, *The water balance of Lake Victoria.* Hydrological Sciences Journal, 1998. **43**(5): p. 789-811.

2.	Fund;, G.N. *Detailed Data Lake Victoria*. 2020 [cited 2020 19.07.].

3.	Pflüger, W., *Experimental epidemiology of schistosomiasis. I. The prepatent period and cercarial production of Schistosoma mansoni in Biomphalaria snails at various constant temperatures.* Z Parasitenkd, 1980. **63**(2): p. 159-69.

4.	Aoki, Y., et al., *Cercariometry for detection of transmission sites for schistosomiasis.* Parasitol Int, 2003. **52**(4): p. 403-8.

5.	Sandt, D.G., *Direct filtration for recovery of Schistosoma mansoni cercariae in the field.* Bulletin of the World Health Organization, 1973. **48**(1): p. 27-34.
